# Early changes in glioblastoma metabolism measured by MR spectroscopic imaging during combination of anti-angiogenic cediranib and chemoradiation therapy are associated with survival

**DOI:** 10.1038/s41698-017-0020-3

**Published:** 2017-06-12

**Authors:** Ovidiu C. Andronesi, Morteza Esmaeili, Ronald J. H. Borra, Kyrre Emblem, Elizabeth R. Gerstner, Marco C. Pinho, Scott R. Plotkin, Andrew S. Chi, April F. Eichler, Jorg Dietrich, S. Percy Ivy, Patrick Y. Wen, Dan G. Duda, Rakesh Jain, Bruce R. Rosen, Gregory A. Sorensen, Tracy T. Batchelor

**Affiliations:** 1 Martinos Center for Biomedical Imaging, Department of Radiology, Massachusetts General Hospital, Harvard Medical School, Boston, MA 02114 USA; 2 Stephen E. and Catherine Pappas Center of Neuro-Oncology, Departments of Neurology, Radiation Oncology, Massachusetts General Hospital, Harvard Medical School, Boston, MA 02114 USA; 3 Medical Imaging Centre of Southwest Finland, Department of Diagnostic Radiology, Turku University Hospital, Turku, Finland; 4 Cancer Therapy Evaluation Program, National Cancer Institute, Bethesda, MD 20892 USA; 5 Center for Neuro-Oncology, Department of Medical Oncology, Dana-Farber Cancer Institute and Harvard Medical School, Boston, MA 02114 USA; 6 Department of Radiation Oncology, Massachusetts General Hospital, Harvard Medical School, Boston, MA 02114 USA; 7 Present Address: Department of Circulation and Medical Imaging, Norwegian University of Science and Technology (NTNU), Trondheim, Norway; 8 Present Address: Department of Nuclear Medicine and Molecular Imaging, University Medical Center Groningen, University of Groningen, Groningen, The Netherlands; 9 Present Address: The Intervention Centre, Clinic for Diagnostics and Intervention, Oslo University Hospital, Oslo, Norway; 10 Present Address: Department of Radiology, University of Texas Southwestern Medical Center, Dallas, TX 75235 USA; 11 Present Address: Brain Tumor Center, Laura and Isaac Perlmutter Cancer Center, New York University Langone Medical Center and School of Medicine, New York, NY 10016 USA; 12 Present Address: Department of Neurology, Maine Medical Center, Portland, ME 04074 USA; 13 Present Address: IMRIS, Deerfield Imaging, Minnetonka, MN 55343 USA

## Abstract

Precise assessment of treatment response in glioblastoma during combined anti-angiogenic and chemoradiation remains a challenge. In particular, early detection of treatment response by standard anatomical imaging is confounded by pseudo-response or pseudo-progression. Metabolic changes may be more specific for tumor physiology and less confounded by changes in blood–brain barrier permeability. We hypothesize that metabolic changes probed by magnetic resonance spectroscopic imaging can stratify patient response early during combination therapy. We performed a prospective longitudinal imaging study in newly diagnosed glioblastoma patients enrolled in a phase II clinical trial of the pan-vascular endothelial growth factor receptor inhibitor cediranib in combination with standard fractionated radiation and temozolomide (chemoradiation). Forty patients were imaged weekly during therapy with an imaging protocol that included magnetic resonance spectroscopic imaging, perfusion magnetic resonance imaging, and anatomical magnetic resonance imaging. Data were analyzed using receiver operator characteristics, Cox proportional hazards model, and Kaplan–Meier survival plots. We observed that the ratio of total choline to healthy creatine after 1 month of treatment was significantly associated with overall survival, and provided as single parameter: (1) the largest area under curve (0.859) in receiver operator characteristics, (2) the highest hazard ratio (HR = 85.85, *P* = 0.006) in Cox proportional hazards model, (3) the largest separation (*P* = 0.004) in Kaplan–Meier survival plots. An inverse correlation was observed between total choline/healthy creatine and cerebral blood flow, but no significant relation to tumor volumetrics was identified. Our results suggest that in vivo metabolic biomarkers obtained by magnetic resonance spectroscopic imaging may be an early indicator of response to anti-angiogenic therapy combined with standard chemoradiation in newly diagnosed glioblastoma.

## Metabolic imaging reveals anti-angiogenic (AA) therapy mechanisms in glioblastoma (GBM)

GBM is the most common and aggressive type of malignant primary brain tumors (glioma). Patients with GBM have less than 5% survival at 5 years with the best existing treatment and progress is desperately needed to improve patient outcome. Imaging can guide development and clinical translation of new treatments, and can help to understand mechanisms of response and to optimize drug regimens in patients. AA therapy that targets the blood supply of tumors can benefit GBM patients as adjuvant therapy to standard chemoradiation, but the timing of combination therapy may be important to obtain the maximum benefit and minimize negative side effects. Tracking metabolic changes with magnetic resonance spectroscopic imaging (MRSI) during a phase II clinical trial of AA drug cediranib combined with chemoradiation shows that there is a therapeutic window within the first month where there is maximal synergy between the effects of the anti-angiogenesis and chemoradiation. Our data suggest that adjusting the dose of AA therapy during chemoradiation may be beneficial, and MRSI can be used as a precision medicine tool to identify the patients and time for adjusting the combination treatment.

## Introduction

Altered metabolism is a hallmark of cancer, which has been recognized as an important mechanism and biomarker for cancer.^1, 2^ In the particular case of glioblastoma (GBM) there is significant alteration in the metabolic profile compared to the healthy brain tissue that can be detected in patients using in vivo magnetic resonance spectroscopy.^3–5^ While initial diagnosis is always confirmed by histopathology and molecular analysis of surgical specimens,^6, 7^ repeated biopsies are not feasible for treatment monitoring in brain tumor patients. On the other hand, imaging can be repeated safely to monitor treatment response, it has the potential to provide objective and precise assessment of response rates in clinical trials, and it could be used to accelerate translation of new therapies. However, conventional anatomical imaging may be ambiguous, often showing signs of pseudo-response (PdR) in the case of angiogenic (AA) therapy or pseudo-progression (PdP) for chemoradiation, respectively.^8–11^ Because of its reliance on biochemistry, magnetic resonance spectroscopic imaging (MRSI) has the potential to address the true/PdR and the true/PdP conundrum. This has motivated us to investigate its value in monitoring newly diagnosed GBM patients treated with adjuvant AA therapy in combination with standard chemoradiation.

Current standard of care for patients with GBM, the most common and aggressive primary brain tumor in adults, is surgical resection followed by fractionated, local radiation, and temozolomide.^12^ Despite aggressive therapy, disease progression is universal and median patient survival is 15 months.^13^ Thus, there is an urgent need to identify therapies that increase GBM patient survival. A fundamental pathophysiological hallmark in GBM is the abnormal tumor vasculature with increased vessel caliber, tortuosity, and permeability^14, 15^ that creates an abnormal tumor microenvironment characterized by hypoxia, acidosis, and elevated fluid pressure which fuels tumor progression and treatment resistance.^16^ These abnormalities are associated with the increased expression of vascular endothelial growth factor (VEGF) and other pro-angiogenic cytokines that stimulate endothelial cell proliferation, migration, and survival.^17^ Compounds that inhibit blood vessel formation, reduce vessel permeability, and alleviate edema have been developed for treating GBM.^18^ Although many of these compounds have promising efficacy in preclinical studies, clinical trials on humans have been less successful showing that only a subset of GBM patients may benefit from AA therapy. Given the adverse effect profile and high cost of AA drugs it is important to predict which patients may benefit and which should avoid AA therapy. Ideally, a method that objectively predicts early the treatment success or failure would have the greatest impact on patient management. The Response Assessment in Neuro-Oncology (RANO) criteria have been specifically designed^19, 20^ to better assess radiological response in glioma patients treated with AA drugs. However, RANO does not fit well with the paradigm of early prediction since it requires late confirmatory scans to rule out PdR/PdP.

Herein, we report results from a prospective longitudinal imaging study of newly diagnosed GBM patients treated with the AA drug cediranib (AstraZeneca), an oral pan-VEGF receptor (VEGFR) tyrosine kinase inhibitor, in combination with standard of care temozolomide and radiation. We hypothesized that metabolic biomarkers acquired by ^1^H MRSI may predict patient outcomes to combination of cediranib with chemoradiation. ^1^H MRSI can reliably detect metabolites such as choline-containing metabolites (total choline, tCho), N-acetyl aspartate (NAA), and creatine (Cr), which generate the dominant signals in brain spectra. A previous study in recurrent GBM patients treated with cediranib monotherapy showed that these metabolites correlated with patient outcomes.^21^ Other studies have found that MRSI is valuable in monitoring temozolomide treatment in glioma patients^22^ and radiotherapy in GBM patients.^23, 24^ However, these studies were performed with a limited number of observations at typical clinical follow-up times, which historically have not been optimized for early detection of treatment response. Our imaging study was designed with the goal of identifying early signs of treatment response. In our imaging protocol we employed weekly scans during the 6 weeks of chemoradiation, which allowed us to closely examine the dynamics of tumor response with unprecedented temporal detail. The goal of our data analysis was to understand the mechanisms of combination treatment, and investigate whether synergistic effects exist between AA therapy and standard chemoradiation.

## Results

Across serial scans the radiological response to therapy was assessed based on changes relative to baseline. In addition to MRSI metabolic changes, other magnetic resonance imaging (MRI) biomarkers such as volume of contrast enhanced T1 (ce-T1), volume of vasogenic edema estimated by FLAIR (Fluid-Attenuated Inversion Recovery) hyperintensity, and relative cerebral blood flow (rCBF) were compared for predicting treatment response. Molecular markers such as MGMT (O^6^-methylguanine-DNA-methyltransferase),^25^ clinical measures such as Karnofsky performance score (KPS) and demographics variables (Age) were assessed in combination with imaging biomarkers.

For MRSI analysis 21 out of 40 patients had data that fulfilled the quality control criteria for longitudinal analysis: (1) consistent positioning of volume of interest (VOI) at baseline and follow-up scans, (2) sufficient spectral quality based on signal to noise ratio (>5), line width (<10 Hz), and (3) absence of major artifacts such as subcutaneous lipid contamination and poorly suppressed water. Across the ten time points the number of patients with analyzable MRSI data varied as follows: 21 at baseline (for both days −5 and −1), 14 at day +1, 12 at day +8, 9 at day +15, 11 at day +22, 21 at day +29, 17 at day +36, 14 at day +43, 12 at day +50. In total we analyzed 162 MRSI data sets, and each MRSI had a VOI that included between 192 and 400 voxels. Missing MRSI follow-up scans were not only due to poor data quality, but mostly because the positioning of the MRSI was not consistent along time points. Often, data were not acquired at some visits in several patients due to time constraints, which accounted for 62% of missing data, or due to patients missing the scan altogether (4%). For the other MRI modalities (ce-T1, FLAIR, rCBF), the number of patients varied between 17 and 21 across the ten scans.

In the group of 21 patients the median progression-free survival (PFS) was 8.8 months (95% confidence interval (CI): 8.2–15.6 months) and median overall survival (OS) was 18.2 months (95% CI: 17.0–27.9 months). Patients were dichotomized into two groups based on their median OS: patients with short OS (<18.2 months), and patients with long OS (>18.2 months).

**Fig. 1:**
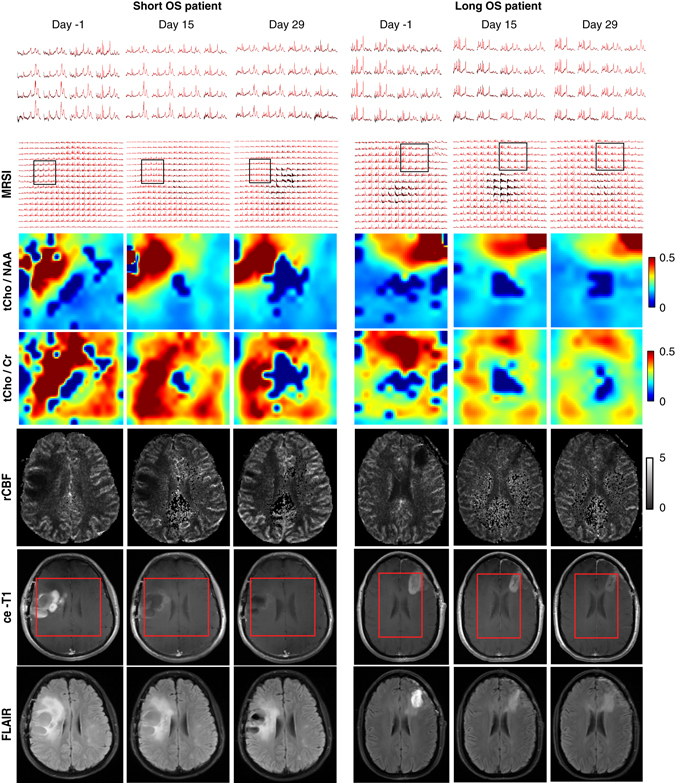
Longitudinal imaging in GBM patients treated with cediranib in combination with standard chemoradiation. Metabolic, perfusion, and anatomical MR images are shown from a patient with short OS (*left*) and long OS (*right*). The short OS patient is representative for PdR with rapid decrease of ce-T1; however, tCho/hCr and tCho/NAA remain elevated. In long OS patient, the decrease of ce-T1 is accompanied by decrease of tCho/hCr and tCho/NAA. Spectra from a region inside tumor are shown on the *upper row*. The *red rectangle* overlaid on ce-T1 images indicates the VOI covered by the MRSI acquisition

In Fig. 1 representative imaging data are shown for a short OS patient and a long OS patient, respectively. In the case of the short OS patient (Fig. 1, *left*) the metabolic maps of tCho/NAA and tCho/healthy creatine (hCr) show a tumor region with elevated choline metabolism that is maintained across time points despite reduction in volumes of ce-T1 and FLAIR, and normalization of tumor rCBF. In the case of the long OS patient (Fig. 1, *right*) there was a significant reduction in levels of both tCho/NAA and tCho/hCr accompanied by normalization (increase) of rCBF. Also, there was a decrease of the tumor region showing elevated choline, ce-T1 uptake, and FLAIR hyperintensity.

**Fig. 2:**
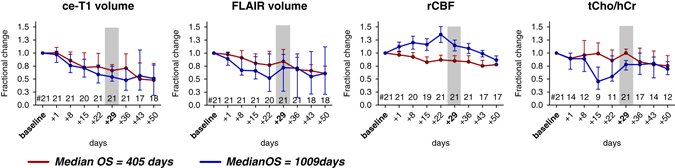
Fractional changes of ce-T1 volume, FLAIR volume, rCBF, and tCho/hCr ratio in GBM patients treated with cediranib in combination with standard chemoradiation. Median time courses in short and long OS patients are shown, and *vertical bars* represent standard error of measurement (s.e.m.)

The median time courses of each imaging biomarker are shown in Fig. 2 for both groups of patients. Out of the 21 patients selected for this analysis, 12 patients had short OS and 9 patients had long OS. The median OS in the short OS group was 405 days, compared with the median OS in the long OS group, which was 1009 days. Figure 2 shows different behavior of imaging biomarkers over time: (i) ce-T1 and FLAIR decrease in both groups, (ii) tCho/hCr is relatively stable in short OS and decreases in long OS patients, (iii) rCBF increases in long OS and decreases in short OS patients. However, two general observations can be made: (1) the changes are larger and faster in long OS patients compared with short OS, (2) the differences between the two groups get smaller at the end of time interval. Of note, there are larger differences between long and short OS patients in the time courses of metabolic–physiological biomarkers tCho/Cr and rCBF, compared with those observed by the common structural biomarkers ce-T1 and FLAIR. The largest differences between short/long OS time courses are noticed between days 15 and 29. Importantly, the median ce-T1 time courses of the two groups are superimposable until day 15, while the other biomarkers diverge earlier. This is important since AA therapy produces early and drastic changes in the appearance of ce-T1, yet these early changes are not specific enough to differentiate short vs. long OS patients. This has potentially important implications for clinical care since ce-T1 is one of the most commonly utilized examinations in brain tumor imaging protocols.

Since the largest differences were noticed within the time interval of +[15–29] days we performed further analysis for these time points, and in particular at day +29 for which we had the same number of patients as in baseline for all imaging modalities.

Cox proportional hazard model was performed to investigate the significant contribution of MR covariates to predict OS. In addition to imaging biomarkers, molecular biomarkers (MGMT), clinical assessment (KPS), and demographics (Age) were also included in the Cox model. Both univariate and multivariate analysis were performed. Table 1 summarizes all the results, including regression coefficients (B), the effect sizes given as hazard ratios (HR = exp(B)), and *P*-values for each of the covariates in relation to OS. In the univariate Cox regressions each covariate was independently assessed and the only statistical significant relation with OS was found for: tCho/hCr (*P* = 0.006), MGMT status (*P* = 0.008), age (*P* = 0.015), and KPS (*P* = 0.032). Decrease of tCho/hCr, positive MGMT methylation, younger age, and higher KPS were associated with longer OS. Importantly, the largest effect size was noticed for tCho/hCr where a unit increase was associated with 86 times increase in HR. The best two imaging biomarkers tCho/hCr (*P* = 0.006) and rCBF (CBF-GE, *P* = 0.108) were combined in a single index tCho/hCr + rCBF. The combined imaging index tCho/hCr + rCBF was also analyzed with a univariate Cox model and was found to have the highest statistical significance (*P* = 0.001). In the multivariate Cox regression we performed two types of analysis. In the first type of multivariate analysis we included two covariates, comparing the imaging index tCho/hCr + rCBF against the other significant covariates MGMT, age, KPS separately. In the bivariate analysis tCho/hCr + rCBF maintains the highest HR and highest statistical significance compared with the other covariates. In the second type of multivariate analysis we included four covariates, using the combined imaging index tCho/hCr + rCBF, FLAIR volume, MGMT status, and age. The combined imaging index tCho/hCr + rCBF was the covariate that was significantly associated with OS and showed a largest effect size. In addition, surgical resection is known to impact survival in GBM patients. However, the majority of patients in our cohort had surgical resections and an analysis of the association between surgery and survival could not be made.

**Table 1:** Cox proportional hazard model of imaging, molecular, clinical, and demographic biomarkers

	Univariate analysis			Multivariate analysis I			Multivariate analysis II		
Variables	B	HR	*P*-value	B	HR	*P*-value	B	HR	*P*-value
Vol-CE	0.63	1.89	0.499						
Vol-FLAIR	0.47	1.60	0.415				0.69	2.01	0.577
CBV-SE	−1.77	0.17	0.171						
CBV-GE	−1.17	0.31	0.133						
CBF-SE	−1.08	0.34	0.267						
CBF-GE (rCBF)	−0.98	0.38	0.108						
tCho/NAA	1.57	4.83	0.158						
tCho/hCre	4.45	85.85	**0.006**						
MGMT	−2.21	0.11	**0.008**	−1.79	0.17	**0.049**	−1.34	0.26	0.216
Age	−0.07	0.93	**0.015**	−0.05	0.96	0.131	−0.03	0.97	0.365
KPS	−3.93	0.02	**0.032**	0.11	1.11	0.958			
tCho/hCr + rCBF	0.39	1.47	**0.001**	0.59	1.79	**0.019**			
				0.72	2.04	**0.001**	0.65	1.91	**0.035**
				0.76	2.14	**0.002**			

*P*-values less than 0.05 are shown in bold

In addition, we also investigated the potential of baseline values of MR parameters, molecular markers, clinical score, and demographics for predicting OS. Results summarized in Supplementary Table 1 show statistical significant association between baseline values and OS in the case of: rCBF (*P* = 0.03), tCho/hCr (*P* = 0.02), volume of ce-T1 (*P* = 0.002), volume of FLAIR (*P* < 0.001), KPS (*P* = 0.032), and Age (*P* = 0.015).

**Fig. 3:**
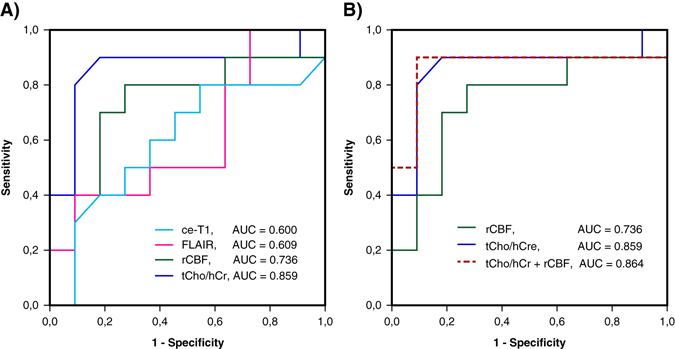
**a** Receiver operating characteristic (ROC) curves for tCho/hCr, FLAIR volume, ce-T1 volume and rCBF. **b** The combined tCho/hCr + rCBF index provides an improvement over tCho/hCr and rCBF alone. Area under curve (AUC) is indicated for each biomarker

Receiver operator characteristic (ROC) analysis was performed using fractional change at day +29 relative to baseline for ce-T1, FLAIR, rCBF, tCho/hCr, and combined tCho/hCr + rCBF index. Fractional changes were based on volumes for ce-T1 and FLAIR, and mean tumor region of interest (ROI) values for rCBF and tCho/hCr. Area under the curve (AUC), threshold, sensitivity, and specificity were determined for all biomarkers. Numerical results of ROC analysis are summarized in Table 2 and graphically plotted in Fig. 3. The highest AUC = 0.864 was obtained for combined tCho/hCr + rCBF index, followed by tCho/hCr with AUC = 0.859, rCBF with AUC = 0.736, FLAIR with AUC = 0.609, and ce-T1 with AUC = 0.6. It is noteworthy that ROC of ce-T1 and FLAIR are only slightly above the line of unity (pure chance), which reflects their diminished value to predict survival and treatment response to anti-VEGFR therapy. Additional ROC analysis performed for baseline values (Supplementary Fig. 1) showed that tCho/hCr at baseline had the highest AUC = 0.836.

**Table 2:** ROC analysis of the imaging biomarkers

Biomarker	AUC	Sensitivity	Specificity	Threshold
ce-T1	0.600	0.60	0.60	0.65
FLAIR	0.609	0.50	0.60	0.85
rCBF (CBF-GE)	0.736	0.80	0.75	1.05
tCho/hCr	0.859	0.90	0.82	0.91
tCho/hCr + rCBF	0.864	0.90	0.91	2.06

Using the thresholds derived from ROC analysis we investigated how each imaging biomarker predicts patient outcome and survival. Patients were split in two groups based on the relative change in the corresponding imaging parameter at day +29 to be below or above the threshold, and the OS was calculated for each group (Supplementary Fig. 2A). It can be seen that the OS values calculated based on tCho/hCr patient stratification were the closest to the true OS values. Using tCho/hCr patient stratification the time courses were calculated for each imaging biomarker (Supplementary Fig. 2B), showing large separation for rCBF and tCho/hCr but overlap for ce-T1 and FLAIR, similar to results of Fig. 2.

**Fig. 4:**
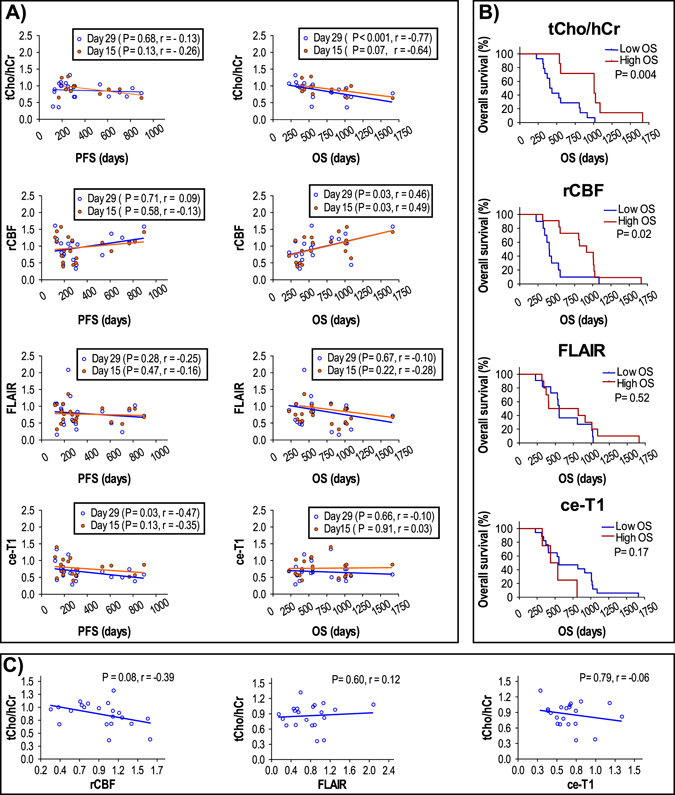
**a** Spearman correlation analysis of imaging biomarkers with PFS and OS at days +15 and +29. **b** Kaplan–Meier log-rank survival plots at day +29 for tCho/hCr, rCBF, FLAIR volume, and ce-T1 volume. **c** Spearman correlation analysis between tCho/hCr and rCBF, FLAIR or ce-T1 at day +29

The fractional change in tCho/hCr, rCBF, FLAIR volume, and ce-T1 volume at days +15 and +29 relative to baseline were further analyzed in relation to PFS and OS duration in Fig. 4. Spearman correlation analysis showed that fractional change of tCho/hCr was negatively correlated with OS at both day +15 (*P* = 0.07, *R* = −0.64) and day +29 (*P* < 0.001, *R* = −0.77). Fractional change of rCBF showed positive Spearman correlation at day +15 (*P* = 0.03, *R* = 0.49) and day +29 (*P* = 0.03, *R* = 0.46). There was a trend for negative correlation between fractional changes of tCho/hCr and rCBF (*P* = 0.08, *R* = −0.39). There was no correlation between fractional changes of tCho/hCr and those of ce-T1 or FLAIR. Volumes of FLAIR and ce-T1 showed no significant correlation with OS for the same time points. With regard to PFS, only ce-T1 volume at day +29 showed a significant negative correlation (*P* = 0.03, *R* = −0.47), which is to be expected since PFS is often decided based on radiological criteria. However, no significant correlation was observed between PFS and OS. Using the ROC-derived thresholds at day +29, log-rank Kaplan–Meier survival curves were determined for all imaging biomarkers. Survival curves obtained by tCho/hCr (*P* = 0.004) and rCBF (*P* = 0.02) were significantly different in short OS and long OS patients, while no significant separation was obtained with ce-T1 and FLAIR.

## Discussion

Imaging has a unique potential to facilitate clinical translation of novel therapies in glioma.^26^ While OS remains the gold standard in evaluating therapeutic benefit, radiographic endpoints that correlate with the clinical endpoint are valuable for providing early prediction of response to therapy. In addition to reducing time and costs of clinical trials, imaging is less confounded by previous treatments in comparison with OS (patient crossover), and can objectively quantify effects of therapy (objective response rate). Currently, PFS, which is typically determined using anatomical imaging, represents a surrogate endpoint used to stratify glioma patients. However historically, anatomical imaging (ce-T1, FLAIR) in GBM patients has shown limited specificity for early anti-tumor treatment effects.^8–11^ Hence, more specific modalities are needed to exploit the full potential of imaging.^27^ Our study provides evidence that supports MRSI as a potentially valuable modality. In particular, we focused on the value of metabolic biomarkers derived from MRSI to monitor patients and assess treatment response.

Assessing response to AA therapy is particularly challenging based on anatomical imaging.^8, 19, 20, 28^ Indeed, data from our current study showed no significant correlation of ce-T1, FLAIR, and PFS with OS. Since AA therapy rapidly restores the permeability of the blood–brain barrier (BBB), reducing contrast leakage and edema probed by ce-T1 and FLAIR, these imaging modalities may become decoupled from the evolution of tumor behind a restored BBB. MRSI, which can probe the tumor metabolic profile irrespective of BBB status, may provide a window into such an invisible tumor during early stages of AA therapy. Such information may be valuable in understanding the mechanisms of AA therapy, its adjuvant role, and how it interacts with concomitant standard chemoradiation.

One hypothesis is that AA drugs act synergistically with standard chemoradiation in GBM patients by promoting functional blood perfusion in the tumor, hence a more efficient delivery of cytotoxic therapy (temozolomide) and oxygen to enhance the genotoxic effects of radiation and chemotherapy.^29–31^ Data from our study showing that a decrease of tumor metabolic activity (tCho/hCr) correlates with increased blood perfusion (rCBF) in responsive patients support this synergistic mechanism early during treatment. The vascular normalization and benefit of AA seems to be transient and limited to the first month of treatment (prior to day +29), resulting in a biphasic response with distinctive early and late behavior seen in the time courses of tCho/hCr and rCBF. Our MRSI data show a transient large decrease in the metabolic activity (tCho/hCr) of tumors between days +15 and +29 in patients that respond to combination of AA and chemoradiation (long OS). However, the difference between metabolic activity of responders (long OS) and non-responders (short OS) decreases toward the end of chemoradiation, which is mirrored by similar behavior of rCBF. This suggests that AA therapy might counter the effect of cytotoxic therapy late in the treatment. Prolonged and high-dose AA therapy may lead to excessive pruning of tumor blood vessels during the late stages of the treatment,^16^ which may explain the late (post day +29) decrease of rCBF in our data (Fig. 2). In addition, decreased permeability of BBB as probed by Ktrans imaging^18, 30^ (shown in Supplementary Fig. 3) may interfere with tumor delivery of drugs such as temozolomide. Likely, there is a delay between early synergistic and late antagonistic effects of AA therapy and these complex dynamics may explain in part why therapeutic benefit has a limited duration.^16^ On the other hand, in non-responsive patients the angiogenesis may not be VEGF-dependent,^14, 16^ hence the blockade of the VEGF pathway will not result in a benefit.

In the cohort of 21 patients treated by the combination of AA and chemoradiation, the tCho/hCr ratio at day +29 was most significantly associated with OS, having the largest AUC and HR as an individual biomarker. The second best imaging biomarker to predict OS was rCBF. Several multiparametric imaging studies showed that combining multiple parameters has superior performance.^30, 32, 33^ We explored this possibility by combining the best two imaging biomarkers tCho/hCr and rCBF. The combined tCho/hCr + rCBF index had a larger AUC than individual biomarkers, and achieved more significance in Cox proportional hazard models compared with other predictors of treatment response such as MGMT methylation status.^25^ The higher AUC of tCho/hCr + rCBF may be explained by the fact that it captures the full effect of combined therapy, tCho/hCr being sensitive to effects of chemoradiation while rCBF probes mostly the effects of AA treatment. We found that MGMT status differed significantly (Mann–Whitney test, *P* = 0.04) between short OS and long OS patients groups, which are stratified based on the cut-off value of tCho/hCr. This association provides a potential mechanism for tCho/hCr sensitivity to chemoradiation effects.

Our results support the possibility that metabolic and physiologic imaging biomarkers can be used to: (1) identify responsive patients, and (2) overcome resistance and improve response to AA therapy.^34^ By monitoring treatment with longitudinal metabolic and physiologic imaging, the dosing regimens can be adjusted during the course of treatment to balance the effects of AA and cytotoxic therapy.

Despite its potential, MRSI is underutilized compared with other MR modalities owing to several limitations. These include suboptimal pulse sequences offered as clinical protocols by vendors, lack of standardization, complex quantification and analysis, as well as difficult integration of anatomical and metabolic information.^35^ These limitations result in less reproducibility and more variability, which lead to less acceptance, fewer studies with smaller number of patients, and less validation. We addressed some of the limitations in our study by employing a custom designed pulse sequence with improved localization, atlas-based positioning of MRSI for improved reproducibility across longitudinal imaging, and spectral fitting based on prior knowledge basis sets.

The small number of patients imposes some limitation on the statistical analysis of our data. In a small cohort only biomarkers that show a large effect size can be tested, multivariate analysis is limited to few variables, and the sampling bias cannot be excluded. However, recent studies^36, 37^ performed with more subjects support the value of MRSI to monitor treatment response in GBM patients. Taken together, our study and others advocate for an earlier imaging investigation time point around the middle of radiotherapy period in GBM. Currently, most clinical protocols have follow-up scans that are farther away in time, after radiotherapy.

In summary, our study may provide information that is relevant to further understand the mechanisms of benefit from AA therapy in GBM, to potentially explain why some patients respond and others fail, the limited duration for which therapeutic benefit is noticed, and the mechanism of action of these agents.^38, 39^ Metabolic profiles obtained by MRSI are complementary to other imaging biomarkers, and our study supports the role of MRSI for comprehensive GBM patient evaluation in future clinical care and in clinical trials.

## Methods

Methods were performed in accordance with relevant guidelines and regulations. Methods were approved by the Internal Review Board committee of Massachusetts General Hospital and Dana-Farber Cancer Institute, Boston, MA.

### Patient eligibility and treatment planning

A cohort of 40 newly diagnosed GBM patients were enrolled in a phase Ib/II clinical trial of cediranib performed concurrently at Massachusetts General Hospital and Dana-Farber Cancer Institute, Boston, MA (ClinicalTrials.gov identifier NCT00662506). All patients participating in this study signed an Internal Review Board approved informed consent form. After 2–3 weeks from surgery, patients received 6 weeks of daily, fractionated radiation with temozolomide. One month following the completion of chemoradiation, temozolomide resumed at 150–200 mg/m^2^ for 6 months. Cediranib was given 30 mg/day orally, starting concomitantly with chemoradiation and continuing without interruption until disease progression or toxicity. Inclusion criteria for all patients were: age ≥18 years with pathology-proven GBM after biopsy or resection who were eligible to receive standard postsurgical temozolomide and radiation, evidence of residual contrast-enhancing tumor ≥1 cm on postsurgical MRI in at least one dimension, a KPS ≥ 60 and Mini-Mental Status Examination score (MMSE) >15, adequate organ and bone marrow function, no concomitant enzyme-inducing anti-epileptic drugs, and no prior anti-VEGF therapy.

### Magnetic resonance imaging

MR data were acquired with a 3.0 T magnetic resonance scanner (TIM Trio, Siemens Healthcare, Erlangen, Germany). The system’s standard 32-channel phased-array head coil was used for imaging. Patients had to be on a stable dose of steroids for 5 days before MRI. The scan time points were performed at days −5 and −1 (baselines) before treatment start, and during treatment at days +1, +8, +15, +22, +29, +36, +43, +50, and monthly thereafter.

MR imaging included anatomical pre-contrast and post-contrast 2D T1-weighted gradient echo (repetition/echo times TR/TE = 600/12 ms, 5 mm slice thickness, 1 mm inter slice gap, 0.43 mm in-plane resolution, 23 slices, 512 × 512 matrix), 2D T2-weighted spin echo FLAIR images (TR/TE = 10,000/70 ms, inversion time TI = 1500 ms, 5 mm slice thickness, 1 mm inter slice gap, 0.43 mm in-plane resolution, 23 slices, and 512 × 512 matrix), 3D T2-SPACE (TR/TE = 3200/428 ms, 1 mm isotropic, 256 × 256 × 176 matrix), post-contrast 3D T1-MEMPRAGE (TI/TR/TE1/TE2/TE3/TE4 = 1200/2530/1.64/3.5/5.36/7.22 ms, 1 mm isotropic, 256 × 256 × 176 matrix). In addition, functional imaging was obtained using dynamic contrast enhanced (DCE), diffusion tensor imaging (DTI), and perfusion-weighted dynamic susceptibility contrast (DSC), as following: DCE—TR = 7.3 ms, TE = 4.4 mm, 2.11-mm slice thickness, 0-mm interslice gap, 20 slices, 1.8-mm in-plane resolution 128 × 128 matrix, field-of-view 230 × 230 mm^2^, bolus of 0.1 mMol/kg injected after 52 s for DCE; DTI—spin-echo EPI, *b*-values = 0/700 s/mm^2^, 42 directions, TR/TE = 7980/84 ms, 64 slices, 1.9 mm isotropic; DSC—combined gradient-echo and spin-echo EPI sequence, TR/TE1/TE2 = 1480/32/93 ms, 5 mm slice thickness, 1.5 mm slice gap, 12 slices, 1.2 mm in-plane resolution, 160 × 160 matrix, 120 frames, a second bolus of 0.1 mMol/kg injected after 80 s for DSC. The contrast injected during DCE served as pre-load dose for DSC scan to minimize effects of contrast agent leakage. Post-contrast T1-weighted images were obtained after the combined DSC + DCE dose of 0.2 mMol/kg of GD-DTPA.

Scan-to-scan reproducibility was improved by the Siemens automatic positioning “AutoAlign” method.^39^ Pre-contrast and post-contrast T1-weighted (ce-T1) and FLAIR images were quantitatively analyzed by an independent neuroradiologist blinded to patient number, clinical data, and treatment status. The lesions were outlined and the volumetric measures were carried out by summing the enhanced post-contrast voxels identified within the lesions. rCBF maps were calculated in nordicICE with post-processing leakage correction and normalization to normal appearing white matter as previously described.^31^ Radiologic tumor response was determined according to the updated RANO criteria for malignant gliomas,^19^ taking into account clinical performance status, dose of steroids, spatial dimensions, and temporal dynamics of contrast-enhancing lesions and FLAIR signal abnormalities. Patients who demonstrated signs of progression on imaging inside the radiation field during the first 12 weeks after completion of chemoradiation were not considered to have tumor progression and (if clinically feasible) were maintained on the same treatment regimen and imaging surveillance. Clinical evaluations were performed by treating the neuro-oncologists and included KPS and MMSE scores.

### Magnetic resonance spectroscopic imaging

2D single-slice MRSI was acquired using localization by adiabatic selective refocusing (LASER) sequence^40^ with acquisition parameters of: TR/TE = 1500/45 ms, FOV = 200 × 160 mm^2^, acquisition matrix 20 × 16, 10 × 10 mm^2^ in-plane voxel, slice thickness 15 mm, NA = 1, elliptical phase encoding, acquisition time TA = 5:02 min:s. LASER selected a VOI, which typically had an in-plane size between 80 × 80 and 100 × 100 mm^2^. Shimming was performed automatically using the method supplied by the manufacturer up to water line width of 15 Hz over the VOI. Water suppression was achieved using the water suppression enhanced through T-1 effects scheme.^41^ AutoAlign method^39^ was used to reproduce consistently the position of MRSI on all visits in a given patient.

MR spectra were fitted with LCModel software^42^ and were further processed and analyzed in MATLAB (Mathworks Inc., Natick, MA, USA). Only voxels for which the goodness of fit as measured by Cramer–Rao lower bounds less than 20% were included in the quantitative analysis. tCho, Cr, and N-acetyl-aspartate (NAA) yielded the largest number of analyzable voxels. Lipid and lactate were overlapping at TE = 45 ms and could not be separated well. Voxel-wise metabolic ratios were calculated for tCho/NAA, while for tCho/Cr the mean value of Cr in the normal appearing white matter (hCr) was first calculated and then used to normalize tCho values in all voxels (further referred as tCho/hCr maps). Mean values of tCho/NAA and tCho/hCr were calculated over the tumor ROI determined on the contrast-enhanced T1-weighted images. Since in some cases the tumor ROI on contrast T1-weighted images disappeared for some of the scans after start of AA treatment, we used the baseline ROI to calculate mean ROI metabolic ratios for all the visits. The region of normal appearing white matter was defined in the contralateral hemisphere. This region included 10–20 MRSI voxels that were selected contiguously in white matter, away from any abnormality and avoiding gray matter and CSF.

### Statistics

Based on the median survival threshold, two groups of histologically confirmed newly diagnosed GBM patients were identified and their clinical parameters, MR imaging, and spectroscopy data were evaluated and compared. To investigate the association between the analyzed MR data and OS and PFS in GBM patients after treatment, Kaplan–Meier curves and log-rank analysis were performed. ROC curve analysis was performed on anatomical MRI and MRSI parameters to establish cut-off values to evaluate the predictive capability of the MR modalities for OS time after 4-week treatment. The AUC for FLAIR volume, ce-T1 volume, rCBF, and tCho/hCr were obtained at 4-week post-treatment time point. Univariate and multivariate Cox proportional hazards model were fit to evaluate any association between MR biomarkers and OS. GraphPad Prism 7 software (GraphPad Software, Inc., La Jolla, CA) was used for statistical analysis.

### Data availability

The data sets generated during and/or analyzed during the current study are available from the corresponding author on reasonable request.

## Electronic supplementary material

Suplementary Information

## Electronic supplementary material

Supplementary Information accompanies the paper on the *npj Precision Oncology* website (doi:10.1038/s41698-017-0020-3).

## References

[1] Cairns RA, Harris IS, Mak TW (2011). Regulation of cancer cell metabolism. Nat. Rev. Cancer.

[2] Agnihotri S, Zadeh G (2016). Metabolic reprogramming in glioblastoma: the influence of cancer metabolism on epigenetics and unanswered questions. Neuro. Oncol..

[3] Nelson SJ (2003). Multivoxel magnetic resonance spectroscopy of brain tumors. Mol. Cancer Ther..

[4] McKnight TR (2004). Proton magnetic resonance spectroscopic evaluation of brain tumor metabolism. Semin. Oncol..

[5] Glunde K, Bhujwalla ZM (2011). Metabolic tumor imaging using magnetic resonance spectroscopy. Semin. Oncol..

[6] Louis DN (2007). The 2007 WHO classification of tumours of the central nervous system. Acta Neuropathol..

[7] Louis DN (2016). The 2016 World Health Organization classification of tumors of the central nervous system: a summary. Acta Neuropathol..

[8] Brandsma D (2009). Pseudoprogression and pseudoresponse in the management of high-grade glioma: optimal decision timing according to the response assessment of the neuro-oncology working group. Curr. Opin. Neurol..

[9] Clarke JL, Chang S, O’Brien BJ, Colen RR (2009). Pseudoprogression and pseudoresponse: challenges in brain tumor imaging. Post-treatment imaging changes in primary brain tumors. Curr. Neurol. Neurosci. Rep..

[10] O’Brien BJ, Colen RR, Brandsma D, van den Bent MJ (2014). Post-treatment imaging changes in primary brain tumors. Pseudoprogression and pseudoresponse in the treatment of gliomas. Curr. Oncol. Rep..

[11] Sorensen AG, Batchelor TT, Wen P, Zhang WT, Jain RK (2008). Response criteria for glioma. Nat. Clin. Pract. Oncol..

[12] Huse JT, Holland EC (2010). Targeting brain cancer: advances in the molecular pathology of malignant glioma and medulloblastoma. Nat. Rev. Cancer.

[13] Ostrom QT (2015). CBTRUS statistical report: primary brain and central nervous system tumors diagnosed in the United States in 2008-2012. Neuro. Oncol..

[14] Jain RK (2007). Angiogenesis in brain tumours. Nat. Rev. Neurosci..

[15] Hanahan D, Weinberg RA (2011). Hallmarks of cancer: the next generation. Cell.

[16] Jain RK (2014). Antiangiogenesis strategies revisited: from starving tumors to alleviating hypoxia. Cancer Cell.

[17] Sitohy B, Nagy JA, Dvorak HF (2012). Anti-VEGF/VEGFR therapy for cancer: reassessing the target. Cancer Res..

[18] Batchelor TT (2007). AZD2171, a pan-VEGF receptor tyrosine kinase inhibitor, normalizes tumor vasculature and alleviates edema in glioblastoma patients. Cancer Cell.

[19] Wen PY (2010). Updated response assessment criteria for high-grade gliomas: response assessment in neuro-oncology working group. J. Clin. Oncol..

[20] van den Bent MJ (2011). Response assessment in neuro-oncology (a report of the RANO group): assessment of outcome in trials of diffuse low-grade gliomas. Lancet Oncol..

[21] Kim H (2011). Serial magnetic resonance spectroscopy reveals a direct metabolic effect of cediranib in glioblastoma. Cancer Res..

[22] Guillevin R (2011). Predicting the outcome of grade II glioma treated with temozolomide using proton magnetic resonance spectroscopy. Br. J. Cancer.

[23] Li Y (2013). Survival analysis in patients with newly diagnosed glioblastoma using pre- and postradiotherapy MR spectroscopic imaging. Neuro. Oncol..

[24] Muruganandham M (2014). 3-Dimensional magnetic resonance spectroscopic imaging at 3 Tesla for early response assessment of glioblastoma patients during external beam radiation therapy. Int. J. Radiat. Oncol. Biol. Phys..

[25] Hegi ME (2005). MGMT gene silencing and benefit from temozolomide in glioblastoma. New Engl. J. Med..

[26] Reardon DA, Ballman KV, Buckner JC, Chang SM, Ellingson BM (2014). Impact of imaging measurements on response assessment in glioblastoma clinical trials. Neuro. Oncol..

[27] Ellingson BM, Bendszus M, Sorensen AG, Pope WB (2014). Emerging techniques and technologies in brain tumor imaging. Neuro. Oncol..

[28] Ellingson BM, Wen PY, van den Bent MJ, Cloughesy TF (2014). Pros and cons of current brain tumor imaging. Neuro. Oncol..

[29] Jain RK (2005). Normalization of tumor vasculature: an emerging concept in anti-angiogenic therapy. Science.

[30] Sorensen AG (2009). A “Vascular Normalization Index” as potential mechanistic biomarker to predict survival after a single dose of cediranib in recurrent glioblastoma patients. Cancer Res..

[31] Batchelor TT (2013). Improved tumor oxygenation and survival in glioblastoma patients who show increased blood perfusion after cediranib and chemoradiation. Proc. Natl. Acad. Sci. U. S. A..

[32] Jalbert, L. E. et al. Magnetic resonance analysis of malignant transformation in recurrent glioma. *Neuro. Oncol*. **23**, 1169–1179 (2016). 10.1093/neuonc/now008 PMC4933480 26911151

[33] Galban CJ (2015). Development of a multiparametric voxel-based magnetic resonance imaging biomarker for early cancer therapeutic response assessment. Tomography.

[34] Lu-Emerson C (2015). Lessons from anti-vascular endothelial growth factor and anti-vascular endothelial growth factor receptor trials in patients with glioblastoma. J. Clin. Oncol..

[35] Oz G (2014). Clinical proton MR spectroscopy in central nervous system disorders. Radiology.

[36] Nelson SJ (2016). Serial analysis of 3D H-1 MRSI for patients with newly diagnosed GBM treated with combination therapy that includes bevacizumab. J. Neurooncol..

[37] Nelson SJ (2017). Association of early changes in 1H MRSI parameters with survival for patients with newly diagnosed glioblastoma receiving a multimodality treatment regimen. Neuro. Oncol..

[38] di Tomaso E (2011). Glioblastoma recurrence after cediranib therapy in patients: lack of “rebound” revascularization as mode of escape. Cancer Res..

[39] Benner T (2006). Comparison of manual and automatic section positioning of brain MR images. Radiology.

[40] Andronesi OC (2010). Spectroscopic imaging with improved gradient modulated constant adiabaticity pulses on high-field clinical scanners. J. Magn. Reson..

[41] Ogg RJ, Kingsley PB, Taylor JS (1994). Wet, a T-1-insensitive and B-1-insensitive water-suppression method for in-vivo localized H-1-NMR spectroscopy. J. Magn. Reson. B.

[42] Provencher SW (2001). Automatic quantitation of localized in vivo 1H spectra with LCModel. NMR Biomed..

