# Supplementary material for: Early changes in glioblastoma metabolism measured by MR spectroscopic imaging during combination of anti-angiogenic cediranib and chemoradiation therapy are associated with survival
Source: NPJ Precis Oncol. 2017 Jun 12;1:20. doi: 10.1038/s41698-017-0020-3 (PMC5708878; doi:10.1038/s41698-017-0020-3)
Supplement: Supplementary file 1 — Suplementary Information [file 41698_2017_20_MOESM1_ESM.docx]

**Supplementary Table 1.** Hazard ratios from the Cox proportional hazard model for baseline values of imaging, molecular, clinical and demographic biomarkers.

|  | **Univariate analysis** | | | | **Multivariate analysis** | | | |
| --- | --- | --- | --- | --- | --- | --- | --- | --- |
| **Variables** | **B** | **HR (exp(B))** | **95% CI for HR** | ***P*-value** | **B** | **HR** | **95% CI for HR** | ***P*-value** |
| CBF-SE | 0.840 | 2.317 | .796-6.748 | 0.123 |  |  |  |  |
| CBF-GE | 0.586 | 1.797 | 1.060-3.047 | 0.030 | -0.536 | 0.585 | 0.110-3.116 | 0.530 |
| CBV-SE | 0.752 | 2.121 | .758-5.728 | 0.138 |  |  |  |  |
| CBV-GE | 0.568 | 1.765 | .989-3.150 | 0.133 |  |  |  |  |
| tCho/hCre | 2.194 | 8.975 | 1.415-56.93 | **0.020** | 4.046 | 57.177 | 1.54-2123.4 | **0.028** |
| tCho/NAA | 1.953 | 7.048 | .185-268.84 | 0.293 |  |  |  |  |
| Vol-CE | 0.026 | 1.026 | 1.01-1.04 | **0.002** | 1.412 | 4.104 | 0.366-46.07 | 0.252 |
| Vol-FLAIR | 0.020 | 1.020 | 1.01—1.03 | **0.000** | -0.276 | 0.759 | 0.153-3.761 | 0.735 |
| KPS | -3.934 | 0.020 | .001-.706 | **0.032** | -2,251 | 0.105 | 0.001-7.514 | 0.301 |
| Age | -0.071 | 0.932 | .880-.987 | **0.015** | -0.069 | 0.933 | 0.877-0.992 | **0.027** |


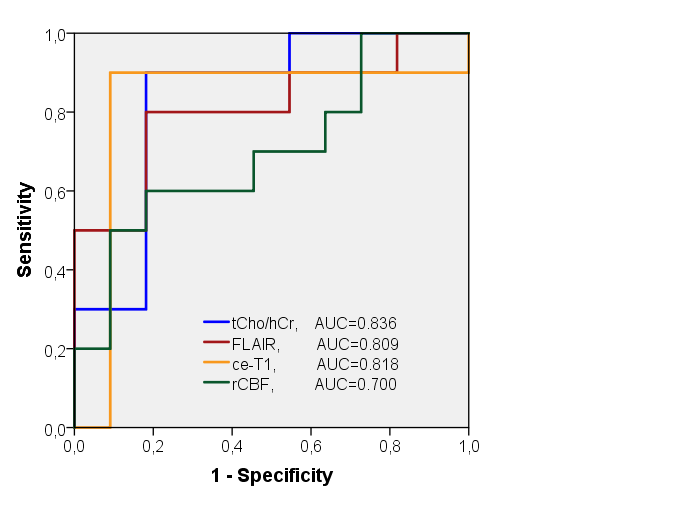


**Supplementary Figure 1.** Receiver operating characteristic (ROC) curves for tCho/hCr, FLAIR volume, ce-T1 volume and rCBF using baseline values. Area under curve (AUC) is indicated for each imaging biomarker.


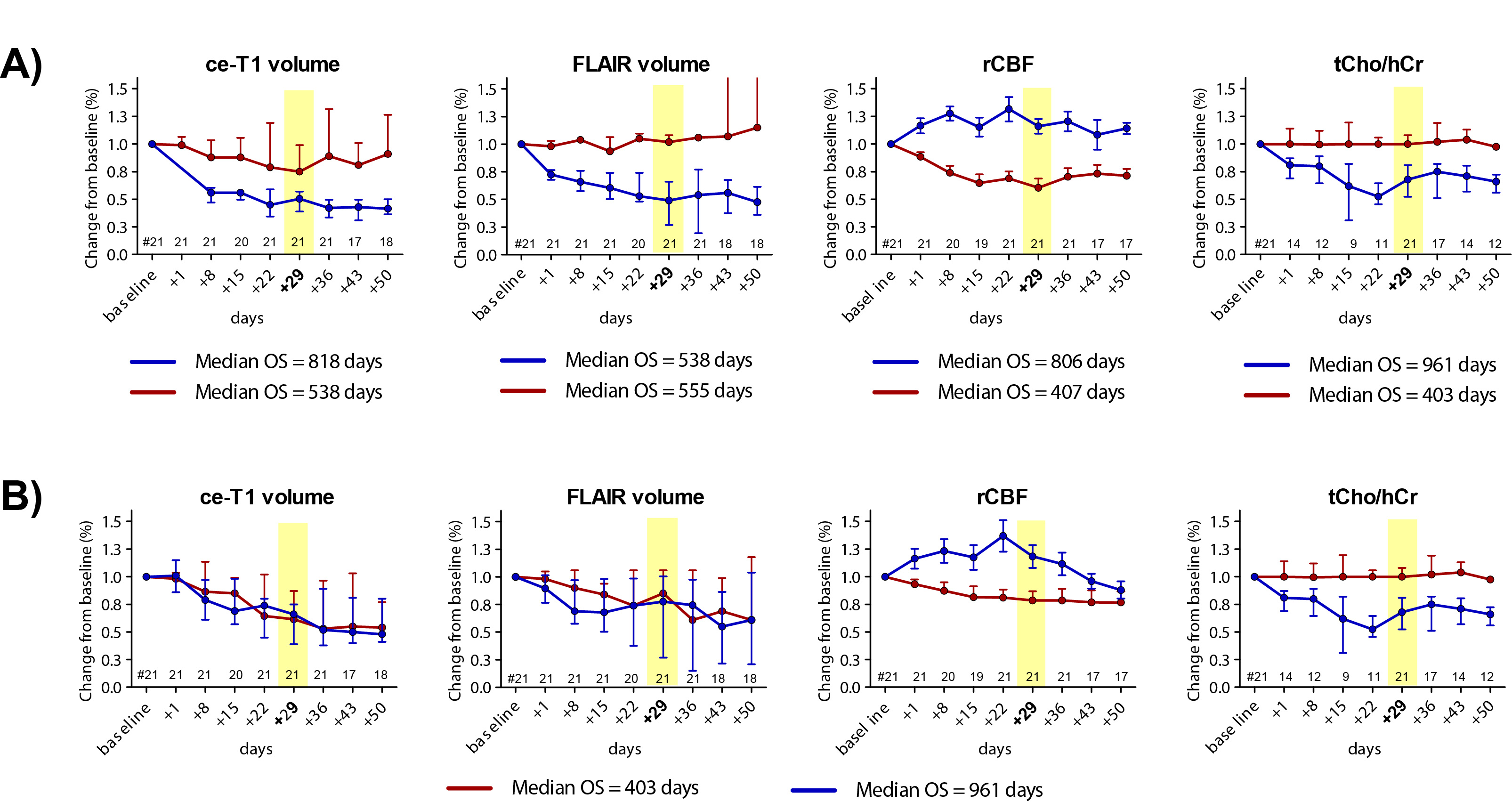


**Supplementary Figure 2.** Time courses of imaging biomarkers during combined antiangiogenic and radiochemotherapy. A) For each imaging biomarker the patients were split in two groups based on the relative change at day +29 as compared to the threshold derived from ROC analysis. For each imaging biomarker the median overall survival in each group was calculated. B) The patient groups obtained using tCho/hCr threshold were used to plot the time courses for all imaging biomarkers. The overall survival times calculated based on tCho/hCr patient groups are nearest to the true OS values, short OS = 405 days and long OS = 1009 days.

**
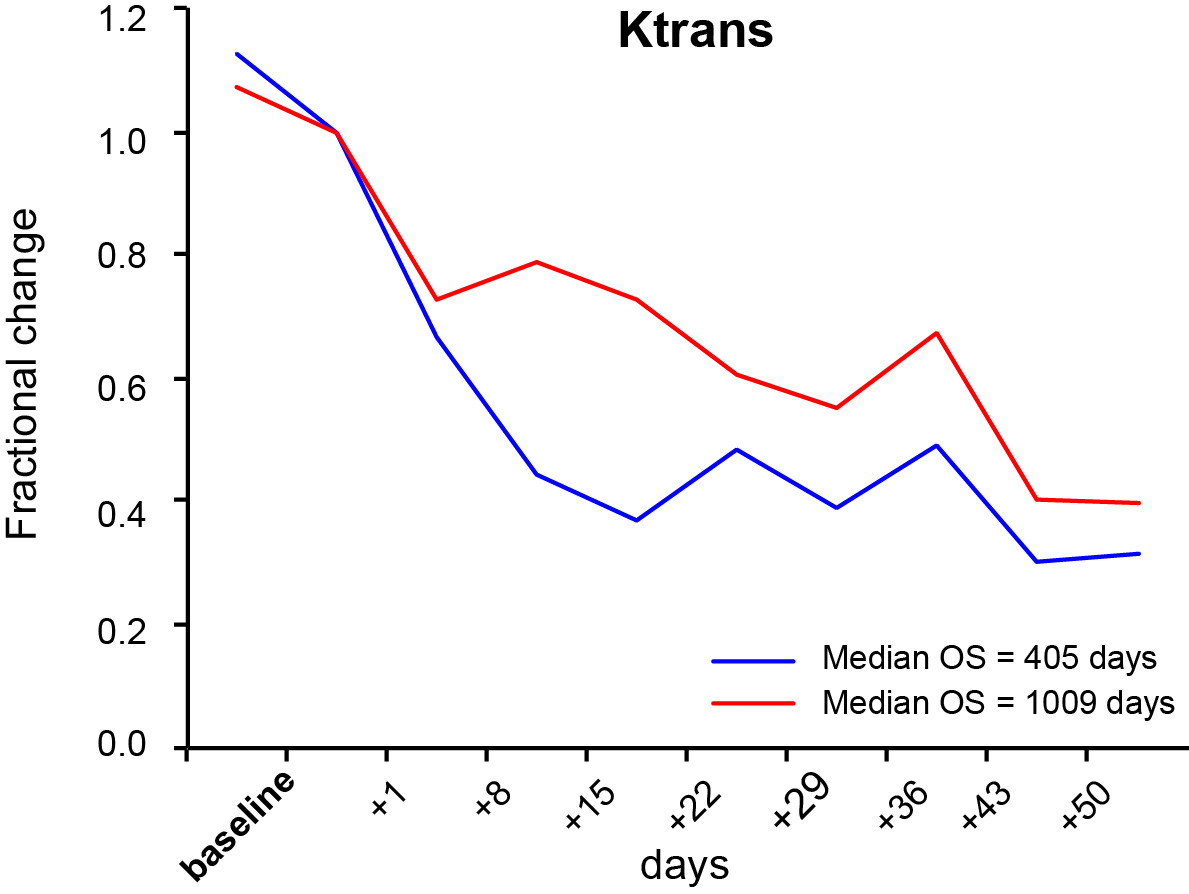
**

**Supplementary Figure 3.** Time courses of Ktrans derived from DCE measurements during combined antiangiogenic and radiochemotherapy. Time courses for the short OS (blue) group and long OS (red). There is more reduction in Ktrans for the short OS group.
